# Pole to Pole Intraocular Transit of Tarantula Hairs—An Intriguing Cause of Red Eye

**DOI:** 10.1155/2009/159097

**Published:** 2009-12-16

**Authors:** Hiten G. Sheth, Patricio Pacheco, Ahmed Sallam, Sue Lightman

**Affiliations:** Department of Clinical Ophthalmology, Institute of Ophthalmology, Moorfields Eye Hospital, City Road, London EC1V 2PD, UK

## Abstract

This intriguing case report provides novel images and a description of the anterior and rarer posterior segment findings seen in ocular inflammation associated with tarantula spider hair exposure. We present an interventional case report of a 9-year-old boy who presented with a red, sore eye. Slit lamp examination revealed right eye injection, multiple small hairs at differing levels of the cornea with associated opacities and inflammation within the anterior and posterior segments of the eye. Only after detailed and repeated questioning did the aetiology become apparent. Conservative management in the form of topical steroid and antibiotics was commenced and he did well with no obvious sequelae in the medium term. Healthcare personnel (and indeed pet shop owners, arachnid enthusiasts and even parents) should be aware of the potential ocular complications of tarantula hair exposure and clinicians should perhaps specifically ask about pet-keeping when presented with an unusual red eye.

## 1. Case Report

**Figure 1:**
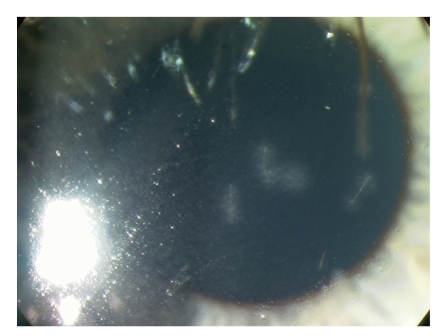
Cornea showing multiple tarantula hairs with associated inflammatory infiltrate and scarring.

**Figure 2:**
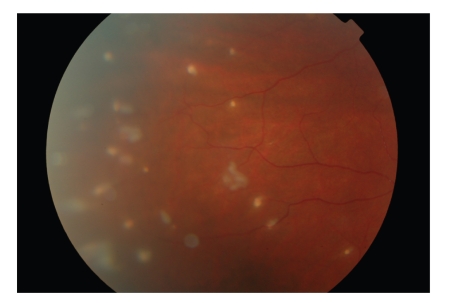
Colour photograph of retina showing punched out full thickness inflammatory lesions associated with embedded tarantula hairs.

A 9-year-old white boy presented to the eye casualty in 2007 with recent onset of a sore, red right eye which had been initially attributed to a play fight 2 weeks previously. Visual acuities were 6/6 (20/20) in both eyes. Slit lamp examination revealed right eye injection, multiple hairs at all levels of the cornea with associated opacities, and a moderate anterior uveitis (Figure 1). Hair-like vitreous lesions and peripheral full-thickness retinal infiltrates were also evident (Figure 2). Left eye examination was normal. After further questioning, it transpired that the boy had a pet Chilean Rose Tarantula spider, thus providing the diagnosis. Topical treatment was commenced with G. Dexamethasone 0.1% qds and G. Chloramphenicol 0.5% qds; he was followed monthly and continued on a long-term tapering regimen of topical steroid. Removal of hairs was felt to be inappropriate in view of the large number of hairs, the deep site of many, and the inability of the patient to tolerate this without general anaesthetic.

At 18-month follow-up both eyes were white and quite with acuities of 6/6 with a successful wean off topical treatment. The corneal hairs had not migrated and were still present in all corneal layers, whilst the retinal lesions were now inactive and less numerous. During this period no complications such as cataract, raised intraocular pressure, macular oedema, or visual loss were observed.

## 2. Discussion

Tarantulas, including the Chilean Rose (Grammostola Rosea), have barbed irritant or urticating hairs as a defence mechanism. These may be shed during casual handling or be thrown off in defence, but either mechanism puts humans at risk of skin, respiratory, and ocular inflammation. Keratitis and iritis, either in isolation or in combination, comprise some of the signs seen in ophthalmia nodosa—the ocular response to a vegetation or animal hair insult [1–4]. Ophthalmia nodosa is so called because of the inflammatory nodular reaction first observed in the palpebral and bulbar conjunctiva, traditionally in response to exposure to barbed caterpillar hairs, and the term is attributed to Saemisch. Due to corneal or even scleral penetration, the condition is increasingly recognised as involving the posterior segment of the eye also following exposure to inflammation-inciting hair or hair-like structures from both insects and plants.

Panuveitis with punctate retinitis is rarer [4, 5] and appears to cause minimal visual morbidity as long as the fovea and macula are spared. Patients are invariably children, presumably because of poor awareness, lack of glove wear and goggle protection, and reduced awareness of hand washing or avoiding rubbing which aids corneal penetration. 

Management includes ocular lubricants, topical steroid to reduce the hypersensitivity reaction and iritis, and early removal of corneal or conjunctival hairs if practical. Choice of steroid drop, frequency of administration, and length of treatment will vary on the visible load of hairs, the associated intraocular inflammatory response, and the subsequent response to treatment. Agents such as dexamethasone 0.1% or prednisolone 0.5% are appropriate, ideally preservative-free. Intraocular surgical intervention is little reported or performed but may be appropriate if there is an intense localised inflammatory response to a focus of hairs in the anterior chamber or vitreous. Laser treatment is not likely to confer any benefit but is used in similarly rare circumstance to treat retinal parasitic worm infections. In summary, management is variable and often difficult and the injury is best avoided via public education.

The barbed arrow-like structure of the spider hairs permits them to penetrate the cornea and enter aqueous and vitreous with ease. This same structure presumably aids retinal entry after which their intra-orbital course is likely to be blocked by the thicker sclera. It remains theoretically possible that they may disseminate systemically via the highly vascularised choroid layer.

Clinicians, parents, and pet-shop owners alike should now be aware of the potential severe ocular complications of tarantula hairs. Clinicians should specifically ask about pet-keeping, including spiders, when presented with an unusual red eye. This case also provides evidence to pet suppliers and parents of the relative unsuitability of spiders as childrens' pets and it is hoped that increasing awareness will help prevent similar cases.
